# Fecal calprotectin Level in patients with IBD and noninflammatory disease of colon: a study in Babol, Northern, Iran

**Published:** 2018

**Authors:** Majid Sharbatdaran, Amin Holaku, Mehrdad Kashifard, Ali Bijani, Alireza Firozjahi, Akram Hosseini, Sepideh Siadati

**Affiliations:** 1 Cancer Research Center, Health Research Institute, Faculty of Medicine, Babol University of Medical Sciences, Babol, Iran; 2 Department of Pathology, Faculty of Medicine, Babol University of Medical Sciences, Babol, Iran; 3 Social determinates Faculty of Medicine, Babol University of Medical Sciences, Babol, Iran

**Keywords:** Fecal calprotectin level, Inflammatory bowel disease, Irritable bowel syndrome.

## Abstract

**Background:**

Inflammatory bowel disease (IBD) is a chronic disease with a relapsing course of inflammation in the digestive system. Endoscopy and histopathology are the golden standard methods for detection and assessment of IBD. A distinct increase of fecal calprotectin level can be a useful marker for the diagnosis of IBD. The aim of this study was to evaluate the fecal calprotectin level in patients with IBD and without inflammatory diseases of the colon.

**Methods:**

Calprotectin levels of patients referred to the Ayatollah Rouhani Hospital of Babol, northern Iran with clinical symptoms of colon disease were evaluated. After a week, colonoscopy and biopsy were performed on all patients and they were divided into two groups. The first group included patients with confirmed IBD and the second group included patients with diseases other than IBD, patients with IBS and healthy persons. Then the measured fecal calprotectin level was compared between the two groups before colonoscopy.

**Results:**

We observed correlation between calprotection in these two groups (p<0.0001). 38 (86.8%) patients in the case group and 5 (13.2%) patients in the control group had positive fecal calprotectin test and 12 (23.1%) patients in the case group and 40 (76.9%) patients in the control group had negative results. Basad on ROC curve, the cutoff point of calprotectin was 127.65 with 73% sensitivity and 89% specificity. The area under the curve was 0.83 with 95% confidence interval, 0.74-0.91 (p<0.0001).

**Conclusions:**

The results pointed to this fact that fecal calprotectin can be a noninvasive marker in differentiating IBD from IBS.

Ulcerative colitis (UC) and Crohn's disease are chronic disorders of the gastrointestinal tract, known as inflammatory bowel diseases (IBD). The symptoms of IBD vary between periods of improvement and flare (1). IBD is more common in developed countries and reaches approximately 1-2 case(s) per 1000 population and its increasing prevalence in both adults and children (2). Due to the common symptoms of IBD with irritable bowel syndrome (IBS) and functional gastrointestinal disorders, the high cost and invasiveness of diagnostic procedures such as endoscopy, barium enema, CT scan and biopsy, low sensitivity and specificity of serologic (CRP) and hematological (ESR) parameters related to the symptoms and signs of IBD, physicians use a combination of clinical signs and symptoms, laboratory indices, radiology, colonoscopy and histopathology to diagnose the disease, assess its severity and predict the outcome (2-5). Among all these methods, endoscopy and histopathology are the golden standard methods in diagnosing IBD (5, 6). 

Using traditional tests is still questionable assuming whether patients with IBD are in recovery or not. Findings of Simren et al. showed that 57% of patients with Crohn's disease and 33% of patients with UC had symptoms like IBS, despite being in a long term recovery period (7). Recently, Keohane et al. confirmed the results of Simren et al. and reported the prevalence of IBS like the symptoms among patients with Crohn's disease and UC was 59.7 and 38.6, respectively (7, 8). Calprotectin is a calcium binding protein, which forms approximately 60% protein content of cytosolic neutrophils and mononuclear cells (9). 

Calprotectin is a protein composed of two heavy chains (L1H) and a light chain (L1L); these two chains are linked together by noncovalent bonds (10-12). Calprotectin is one of the most important regulatory proteins in the inflammatory response (13-15). Studies on children and adults have shown that there was a correlation between fecal calprotectin and the severity of mucosal inflammation (16, 17). Since it is not possible to differentiate IBD from IBS in many cases, using a marker is justified to differentiate the two diseases. Although the extensive serological research studies have been done to differentiate IBD from IBS in recent years, there has been little success (18). 

Significant increase of fecal calprotectin levels is considered as a useful marker for the diagnosis of intestinal inflammation among the laboratory parameters due to its low cost and easy to measure (approximately 5 g), excellent stability at room temperature for a week and the ability to be examined using ELISA immunoassay kits (5, 6). Roseth et al. Schoepfer et al. and Keohane et al. have approved some hopeful results when they measured the fecal calprotectin (zinc binding protein) (8, 9, 18). 

The aim of this study was to evaluate the level of fecal calprotectin in patients with IBD and patients without inflammatory diseases of the colon.

## Methods

The present case-control study was conducted on patients referred to a gastroenterologist in Ayatollah Rouhani Hospital of Babol, northern Iran with clinical symptoms of colon diseases during 2013-2014. Participants were older than 18 years, which include both genders. All patients had an indication for colonoscopy, which was diagnosed by gastroenterologist. The exclusion criteria included suffering from any chronic disease and illness that caused fever in patients. Sampling method was relatively easy and the information of the patients such as age, gender, education and occupation was written in the form. Finally, 90 patients were entered into this study. First, the calprotectin levels of all patients were measured and recorded. Calprotectin test was performed using ELISA method by the Buhlmann Laboratories Kit made in Switzerland. Calprotectin less than 50μg is considered negative and more than 200μg is considered positive. Patients with calprotectin level of 50-200μg were excluded from the study, however follow-up was done.

The colonoscopy was performed on all patients one week after their calprotectin was measured. During colonoscopy, three biopsies were taken from the most severe local inflammation and were sent to pathology department. Then 40 patients diognosed with IBD were divided into two groups (45 per group) according to the results of the colonoscopy and biopsy. The first group included patients with confirmed IBD and the second or control group included patients with inflammatory bowel diseases other than IBD, patients with IBS and healthy persons. After that, the measured fecal calprotectin levels before colonoscopy were compared in two groups.

Data were analyzed with SPSS Version 22 software and ROC curve. Area under the ROC curve was considered as diagnostic value of calprotectin and with the 95% confidence interval. Also, using the ROC curve, sensitivity and specificity of cutoff point was calculated. Therefore reduced false positive and false negative, Mann-Whitney, chi-square and t-test were used for quantitative and qualitative variables. A p-value<0.05 was considered significant.

## Results

The mean age of patients was 34.69±10.42 years. Totally, 39 (43.3%) males and 51 (56.7%) females participated in the present study (table 1). There was significant result between calprotectin and both groups (p<0.0001), thus 33 (73.3%) patients in the case group and 5 (11.1%) patients in the control group had positive fecal calprotectin test while 12 (26.7%) patients in the case group and 40 (88.9%) patients in the control group had negative results. The diagnostic value of calprotectin in comparison with biopsy was evaluated. According to the ROC curve, cutoff point of calprotectin was 127.65 with 73% specificity and 89% sensitivity, respectively (table 2).

**Table 1:** Comparison of mean of age, fecal calprotectin and sex distribution between IBD patients and control subjects

**Pvalue**	**Case**	**Control**	**Group** **Variables**
0.779	34.38±11.30	35±9.59	Mean age (year)
			**Calprotectin**
0.000	652.8±799.7 315.9 (48.3-843.5)	98.30±256.1 42 (32-47.6)	Meam±SD Medican (IQR)
			**Gender**
0.395	22 (48.9) 23 (51.1)	17 (37.8) 28 (62.2)	Men (%) Women (%)

**Table 2:** Diagnostic precision of calprotectin in IBD

**Diagnostic precision**	**Value**	**95% CI**
Sensitivity	73%	(60%-86%)
Specificity	89%	(80%-98%)
Positive predictive value	87%	(76%-98%)
Negative predictive value	77%	(65%-88%)
Likelihood ratio+	6.60	(2.84-15.36)
Likelihood ratio-	0.30	(0.18-0.49)

**Figure 1:**
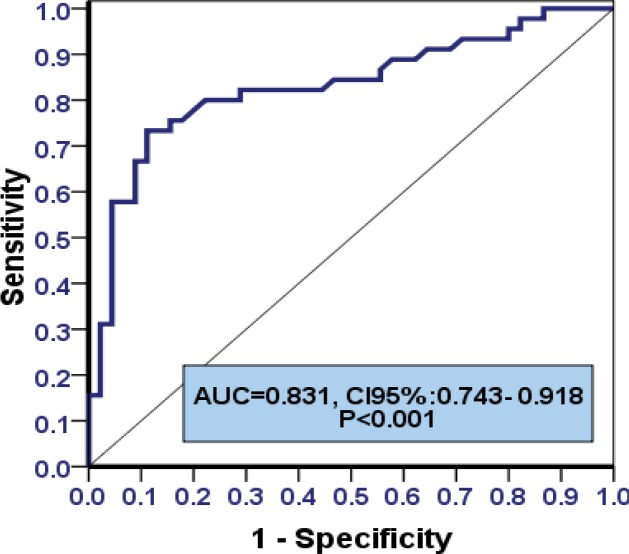
ROC curve showing the correlation between specificity and sensitivity of calprotectin in the diagnosis of IBD

Areas under the curve was 0.83 with 95% confidence interval, 0.74-0.92 (p<0.0001) (figure1).

## Discussion

Based on the results, the cutoff point of calprotectin was 127.65 with 73% specificity and 89% sensitivity. The average of calprotectin in case group was higher than the other group. Keohane et al. (8) found a significant correlation between the increased calprotectin levels in both the IBS and IBD patients; Schoepfer et al. (18) expressed that the fecal level of this marker can differentiate IBD from IBS with high sensitivity and accuracy, which was consistent with the results of the present study. Moein et al. demonstrated that FC has better effect in the differentiation between the subjects with IBD from those without IBD than conventional inflammatory marker (19).

A study in Ireland stated that the combination of clinical symptoms with noninvasive markers such as calprotectin was very important (8). Although using the laboratory markers with this sensitivity is not necessary, these tools can be useful (20). On the other hand, various levels of calprotectin have been described in several studies to differentiate IBD from IBS. Tibble et al. reported that 30 mg/g level had 100% sensitivity to differentiate IBD from IBS (21). Also, D' Inca et al. and Sipponen et al. suggested 130 and 200 microgr/gr levels for activating and improving the disease, respectively (22, 23). In a recent study published by Sipponen et al. the best value has been considered 94 micrograms per gram. Thus, calprotectin levels can be helpful in differentiating IBD from IBS (24).

Some studies have suggested that fecal calprotectin level is significantly higher in some intestinal disorders (including esophageal / gastric carcinoma, Crohn's disease, ulcerative colitis and colorectal carcinoma) than other disorders (Barrett's esophagus, stomach ulcers, gastritis / duodenitis, colorectal polyps and adenoma) (25). In this study, only IBD was compared to other inflammatory bowel diseases and there was no information on other diseases in the control group. Besides, the ROC curve analysis confirmed the fact that fecal calprotectin level was significantly higher among the IBD patients and other individuals (healthy or suffering from IBS) and the increase of calprotectin level is associated with the increase of IBD risk.

The strength of the study presented two views of calprotectin. First calprotectin was considered as an inflammatory factor, then using the ROC curve, it was considered as a diagnostic marker. Limitations of this study include lack of evaluating and identifying non-inflammatory bowel diseases (in the control group). Moreover, the relationship between fecal calprotectin and IBD was generally compared, but the Crohn's disease and ulcerative colitis were not separately studied. In summary, the results showed that fecal calprotectin could be a noninvasive marker to differentiate IBD from IBS. Considering the cutoff point of calprotectin with 73% sensitivity and 89% specificity, it could be used as a diagnostic method to differentiate the inflammatory bowel diseases.
